# Analysis of disease burden of osteoarthritis in China from 1990 to 2021 and projections for 2022–2044

**DOI:** 10.1016/j.ocarto.2026.100859

**Published:** 2026-08-10

**Authors:** Binni Yang, Zhe Wang, Wantian Feng, Chen Gao

**Affiliations:** a Department of General Practice, The 940th Hospital of Joint Logistics Support Force of Chinese People's Liberation Army, Lanzhou, Gansu, China; b First School of Clinical Medical, Gansu University of Chinese Medicine, Lanzhou, Gansu, China

**Keywords:** Osteoarthritis, Disease burden, Prediction, Disease prevention, China

## Abstract

**Objective:**

To analyze the secular trend of osteoarthritis (OA) disease burden in China from 1990 to 2021 and predict future burden changes spanning 2022–2044 to support targeted public health intervention.

**Methods:**

Based on the Global Burden of Disease 2021 (supplemented with partial 2023 GBD data), crude incidence, age-standardized incidence rate (ASIR), disability-adjusted life years (DALYs) and OA prevalence data were retrieved. Joinpoint regression calculated average annual percentage change (AAPC) to assess secular trends; ARIMA and Brown time-series models were selected via AIC and residual diagnosis for future burden prediction, with 95% forecast uncertainty intervals calculated.

**Results:**

National OA prevalent cases increased from 4.65 million in 1990 to 11.65 million in 2021, with the AAPC of ASIR and age-standardized DALY rate reaching 0.43% and 0.49% (all P < 0.001). China's ASIR surpassed the global average after 2003; females sustained higher OA burden than males, incidence peaked among people aged 55–59 years, and DALY loss predominantly clustered in the population ≥85 years. Knee OA dominated the burden across all anatomical subtypes. Forecasts indicate the national ASIR and standardized DALY rate will rise to 589.00/100,000 and 292.02/100,000 by 2044 with inherent predictive uncertainty.

**Conclusion:**

China's overall OA burden has risen substantially and will keep increasing over the next two decades. Females, middle-aged and elderly residents, and patients with knee OA constitute core high-risk groups. These findings provide reliable epidemiological evidence for hierarchical OA prevention and national health policy formulation.

## 1 Introduction

Osteoarthritis (OA) is a prevalent chronic degenerative joint disease mainly involving weight-bearing joints including knees, hips, spine and hands [1]. Persistent joint pain, stiffness and progressive articular deformity impair daily mobility and may eventually lead to permanent disability, with reported disability rate up to 53% [2]. Chronic physical discomfort also raises risks of depression and anxiety and impairs patients’ quality of life. Global OA prevalence keeps climbing amid population aging and brings mounting socioeconomic burdens worldwide [3].

OA-related irreversible articular damage determines the necessity of early intervention; however, limited early diagnostic criteria, insufficient public cognition and imperfect prevention policies hinder standardized disease control [4]. Previous Chinese OA burden analyses were mostly based on outdated GBD datasets before 2017. To update epidemiological evidence, the present study used GBD 2021 data to quantify China's OA burden from 1990 to 2021 stratified by age, gender and pathological subtype, alongside long-term burden prediction for 2022–2044. Findings will facilitate targeted health promotion, reduce OA-related disability and ease corresponding social economic costs.

## 2 Materials and methods

### 2.1 Data source

The study data were mainly extracted from the GBD 2021 database, which evaluates the disease burden caused by 369 diseases and 87 risk factors in 204 countries, with detailed information on age, gender, region, and other dimensions. Data on OA attributed to smoking-induced digestive system diseases in the GBD 2021 database are mainly derived from death surveillance systems, vital registration systems, and the Chinese Center for Disease Control and Prevention [[5], [6], [7], [8]]. For this study, we extracted data on crude incidence rate, age-standardized incidence rate (ASIR), and disability-adjusted life years (DALYs) of OA in China from 1990 to 2021 to analyze the disease burden over this period. The data for this study were accessed on September 10, 2025. During the data extraction process, the authors did not have access to any personally identifiable images or data that could identify individual participants. The GBD database has released its 2023 updated version recently; however, official full public access to China-specific 2023 OA stratified data (age, gender, joint subtypes) is currently unavailable.

### 2.2 Statistical methods

#### 2.2.1 Time trend analysis

The Joinpoint regression model (Joinpoint [4.2.0.1](4.2.0.1)) was used to assess the time trend of OA disease burden in China. This model divides the entire study period into segments and identifies time points where significant trend changes occur. The annual percentage change (APC) and its 95% confidence interval were estimated for each segment, and the average annual percentage change (AAPC) and its 95% confidence interval were calculated for the entire period using a log-linear model. A permutation test was used to determine whether the APC of each segment was statistically significant, and the optimal model recommended by the Joinpoint regression program was adopted [[9], [10], [11]]. Set maximum allowed joinpoint numbers per regression based on sample size following standard epidemiological practice. An APC or AAPC >0 indicates an increasing trend, while a value < 0 indicates a decreasing trend. The significance level was set at α = 0.05 [12].

#### 2.2.2 Autoregressive Integrated Moving Average (ARIMA) model

The ARIMA model is a popular and powerful method for time series prediction, combining the “memory” feature of autoregressive (AR) models, the stationarization capability of differencing (I), and the error term processing capability of moving average models. Before constructing ARIMA models, Augmented Dickey–Fuller (ADF) stationarity tests were performed on original time series to judge whether differencing transformation was required. The NINIC procedure in SAS software was used for optimal model order selection [13,14]. The upper limit of autoregressive order *p* and moving average order *q* was set to 5 to avoid overfitting. The values of p and q were determined based on autocorrelation and partial autocorrelation plots, and the optimal model was selected according to the minimum Akaike Information Criterion [15]. To further evaluate model adequacy, Ljung-Box white noise tests were conducted to detect residual autocorrelation; ten-fold cross-validation was also adopted to calculate out-of-sample mean absolute percentage error (MAPE) and assess generalization ability. Only models with Ljung-Box P > 0.05 and low cross-validation MAPE were retained for long-term extrapolation. The significance level was set at α = 0.05.

#### 2.2.3 Brown's linear exponential smoothing

The Brown model was selected for projecting the Age-Standardized Incidence Rate (ASIR). Unlike the DALYs rate, the incidence trend exhibited a stable linear pattern without significant fluctuations. Brown's linear exponential smoothing is statistically optimal for forecasting data with a linear trend. Given the long-term projection horizon (20 years), this method provides more stable and robust estimates for such a consistent trend compared to ARIMA models, which are more sensitive to short-term stochastic variations.

## 3 Results

### 3.1 Disease burden of osteoarthritis in China

**Fig. 1:**
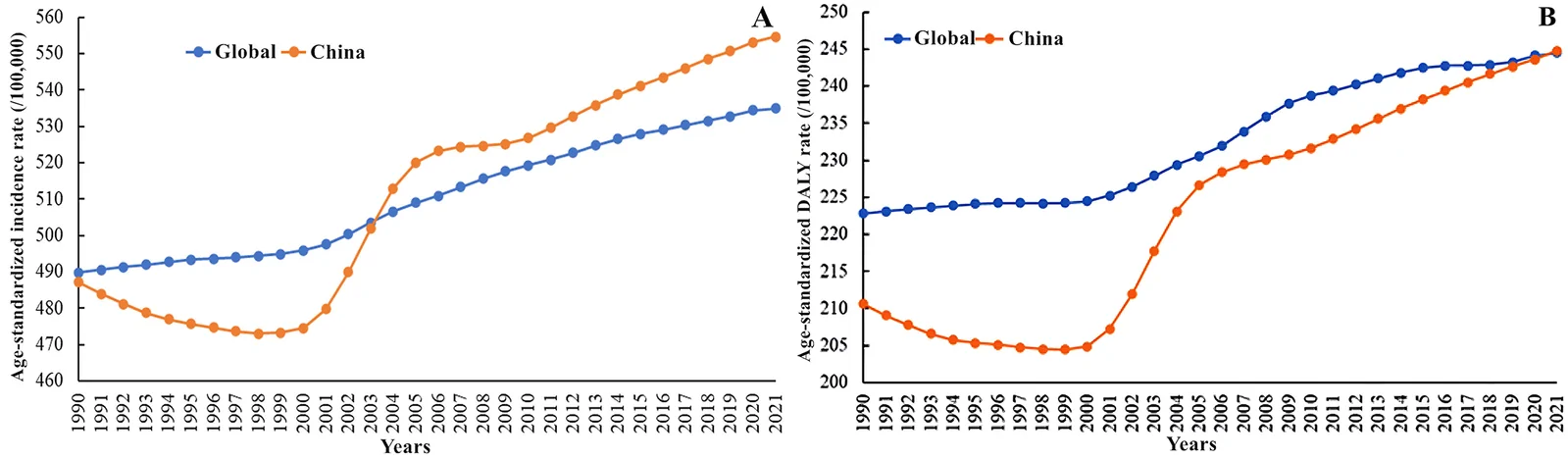
Burden of Osteoarthritis in China and Globally, 1990 and 2021. (A) Age-standardized incidence rate (/100,000). (B) Age-standardized DALY rate (/100,000).

In 1990, the number of OA cases in China was 4.6541 million, with a crude incidence rate of 395.61 per 100,000. The DALYs were 1.8294 million person-years, and the crude DALY rate was 155.50 per 100,000. By 2021, the number of OA cases had increased to 11.6527 million, with a crude incidence rate of 819.03 per 100,000. The DALYs reached 5.3274 million person-years, and the crude DALY rate was 374.44 per 100,000. From 1990 to 2019, the age-standardized incidence rate and standardized DALY rate showed an increasing trend, and the OA disease burden in China in 2021 was significantly higher than that in 1990 (Table 1, Fig. 1).

**Table 1:** Burden of osteoarthritis in China and globally, 1990 and 2021.

Indicator	China						Global					
	1990			2021			1990			2021		
	Female	Male	Total	Female	Male	Total	Female	Male	Total	Female	Male	Total
Number of cases (10,000)	275.29	190.13	465.41	698.11	467.17	1165.27	1239.67	850.38	2090.05	2770.27	1892.95	4663.21
Crude incidence rate (/100,000)	483.28	313.31	395.61	1004.99	641.62	819.03	468.18	316.63	391.86	704.55	478.09	590.93
Age-standardized incidence rate (/100,000)	589.07	389.19	487.11	664.05	445.31	554.61	568.60	408.56	489.78	621.32	445.74	535.00
DALYs (10,000 person-years)	109.72	73.23	182.94	323.25	209.49	532.74	548.52	343.37	891.89	1302.13	828.33	2130.46
Crude DALY rate (/100,000)	192.61	120.67	155.50	465.35	287.71	374.44	207.15	127.85	167.22	331.16	209.21	269.97
Age-standardized DALY rate (/100,000)	250.86	168.91	210.61	290.18	197.25	244.79	257.65	182.42	222.80	284.14	200.52	244.50

### 3.2 Global disease burden of osteoarthritis

In 1990, the global number of OA cases, DALYs, crude incidence rate, and crude DALY rate were 20.9005 million, 8.9189 million person-years, 391.86 per 100,000, and 167.22 per 100,000, respectively. By 2021, these indicators had risen to 46.6321 million, 21.3046 million person-years, 590.93 per 100,000, and 269.97 per 100,000. From 1990 to 2021, the standardized DALY rate and incidence rate of OA in China showed a significant upward trend: the standardized incidence rate in China was lower than the global level before 2003 but higher than the global level after 2003, while the standardized DALY rate in China was lower than the global level before 2021 (Table 1, Fig. 1).

### 3.3 OA disease burden by gender in China

**Fig. 2:**
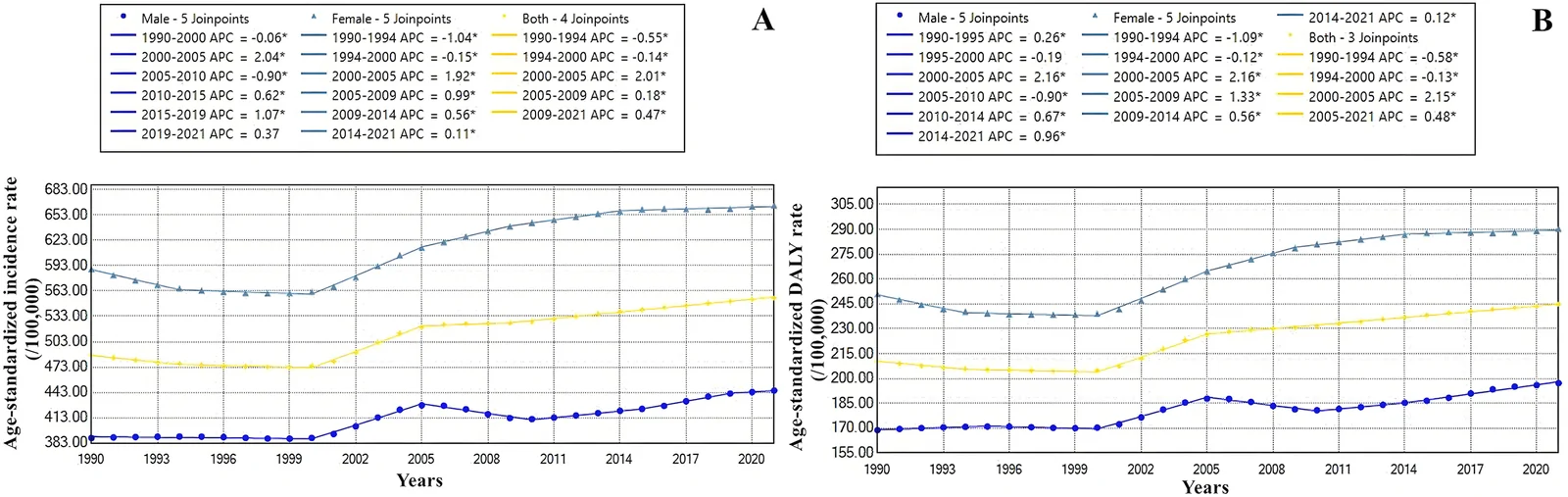
Disease Burden of Osteoarthritis by Gender in China, 1990–2021. (A) Age-standardized incidence rate (/100,000). (B) Age-standardized DALY rate (/100,000).

From 1990 to 2021, the age-standardized incidence rate of OA in males increased from 389.19 per 100,000 to 445.31 per 100,000, and the standardized DALY rate increased from 168.91 per 100,000 to 197.25 per 100,000. In females, the standardized incidence rate rose from 589.07 per 100,000 to 664.05 per 100,000, and the standardized DALY rate increased from 250.86 per 100,000 to 290.18 per 100,000. The standardized incidence rate and DALY rate in females were higher than those in males in all years (Table 1, Fig. 2). Overall, the standardized incidence rate and DALY rate of OA showed an upward trend from 1990 to 2021, with AAPC values of 0.43% and 0.49%, respectively (both *P* < 0.001).

For males, the AAPC of the standardized incidence rate was 0.42% (*P* < 0.001), showing an increasing trend over the entire period. By segment: it decreased from 1990 to 2000 (APC <0, P < 0.001), increased from 2000 to 2005 (APC = 2.40, *P* < 0.05), decreased from 2005 to 2010 (APC = −0.90, *P* < 0.05), and increased again from 2010 to 2021 (APC >0, *P* < 0.05). For females, the AAPC of the standardized incidence rate was 0.39% (*P* < 0.001), with a decreasing trend from 1990 to 2000 (APC <0, *P* < 0.05) and an increasing trend from 2000 to 2021 (APC >0, *P* < 0.05).

The AAPC of the standardized DALY rate in males was 0.51% (*P* < 0.001), showing an overall upward trend: increasing from 1990 to 1995 (APC = 0.26, *P* < 0.05), decreasing from 1995 to 2000 (APC = −0.19, *P* < 0.05), increasing from 2000 to 2005 (APC = 2.16, *P* < 0.05), and increasing again from 2010 to 2021 (APC >0, *P* < 0.05). In females, the AAPC of the standardized DALY rate was 0.47% (*P* < 0.001), with a decreasing trend from 1990 to 2000 (APC <0, *P* < 0.05) and an increasing trend from 2000 to 2021 (APC >0, *P* < 0.05) (Fig. 2, Table 2).

**Table 2:** Trends in osteoarthritis burden by joinpoint regression.

Indicator	AAPC (95% CI)	*t*-value	*P*-value
Age-standardized incidence rate			
Male	0.42 (0.36–0.48)	13.6	<0.001
Female	0.39 (0.34–0.43)	17.08	<0.001
Total	0.43 (0.39–0.46)	24.28	<0.001
Age-standardized DALY rate			
Male	0.51 (0.43–0.59)	12.86	<0.001
Female	0.47 (0.42–0.52)	18.84	<0.001
Total	0.49 (0.46–0.52)	31.54	<0.001

### 3.4 OA disease burden by age in China

The incidence rate of OA increased with age: it remained low in populations under 39 years old, began to increase slowly in those aged 40–54, reached the highest the 55–59 age group, and remained stable in those aged 55–85 and above. The DALY rate of OA was mainly concentrated in people over 55 years old, with the highest DALY rate in the age group over 85 years old.

**Fig. 3:**
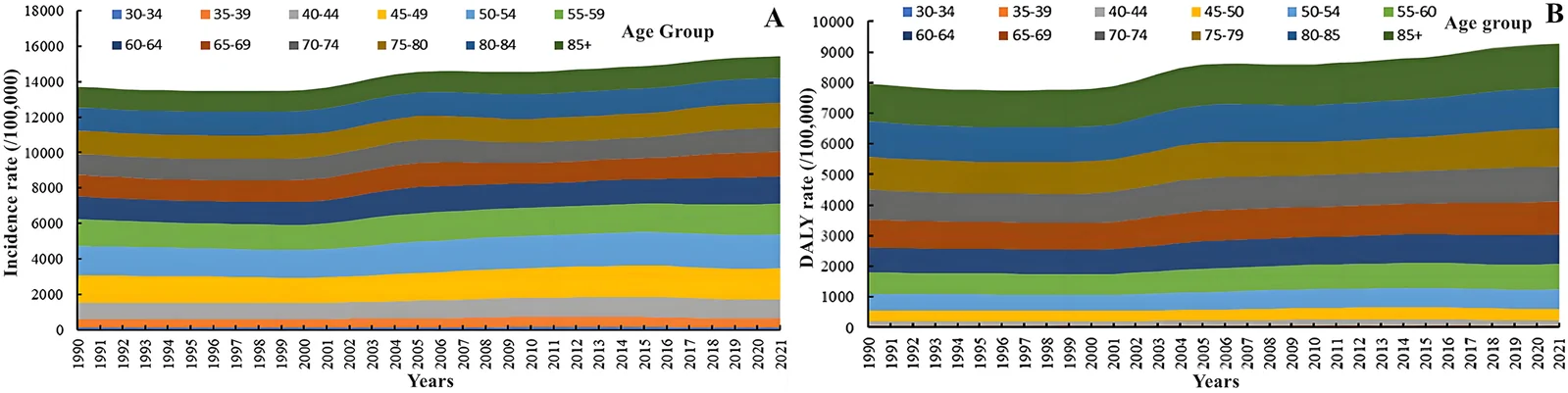
Disease Burden of Osteoarthritis by Age Group in China, 1990–2021. (A) Incidence rate (/100,000). (B) DALY rate (/100,000).

From 1990 to 2004, the age-specific incidence rate and DALY rate of OA remained stable across all age groups; after 2004, these indicators began to gradually increase (Fig. 3).

### 3.5 Disease burden of OA subtypes in China

**Fig. 4:**
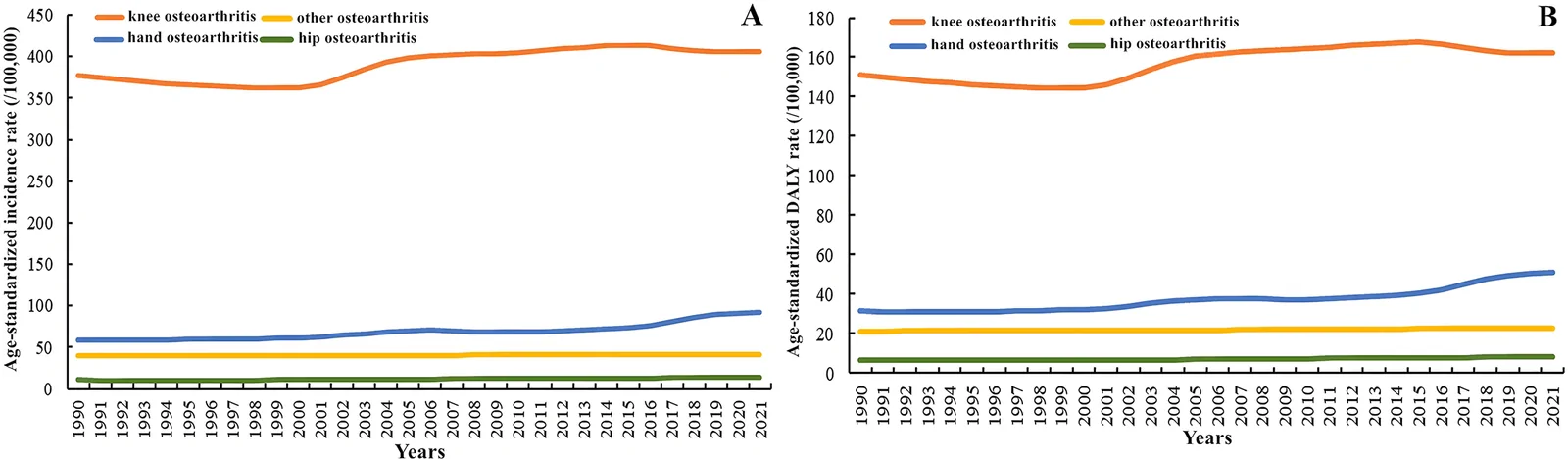
Disease Burden of Osteoarthritis Subtypes in China, 1990–2021. (A) Age-standardized incidence rate (/100,000). (B) Age-standardized DALY rate (/100,000).

In the GBD 2021 database, OA subtypes mainly include hand osteoarthritis, hip osteoarthritis, knee osteoarthritis, and other osteoarthritis. From 1990 to 2021, the standardized incidence rate and DALY rate of knee osteoarthritis were significantly higher than those of other subtypes, while hip osteoarthritis had the lowest standardized incidence rate and DALY rate. In 2021, the standardized incidence rate and standardized DALY rate of knee osteoarthritis were 406.42 per 100,000 and 162.44 per 100,000, respectively; for hand osteoarthritis, the rates were 92.70 per 100,000 and 51.26 per 100,000; for hip osteoarthritis, the rates were 0.62 per 100,000 and 13.86 per 100,000; and for other osteoarthritis, the rates were 41.63 per 100,000 and 22.75 per 100,000. Compared with 1990, all burden indicators of OA subtypes in China increased in 2021 (Fig. 4).

Interpretation of Hip OA Data: It is important to note that the exceptionally low recorded incidence rate of hip OA may not fully reflect the true epidemiological burden. We tentatively put forward a hypothesis that this anomaly may stem from significant underdiagnosis in primary care settings and limited access to diagnostic imaging (e.g., X-ray, MRI) in resource-constrained regions of China. Unlike knee OA, which presents with more obvious symptoms, hip OA is often misdiagnosed or overlooked in the early stages. However, this explanation remains speculative without supporting clinical registry or imaging prevalence data from Chinese populations, and should not be regarded as a definite conclusion; the reported incidence may only represent the ‘tip of the iceberg’ of the actual disease burden.

### 3.6 Model construction and projection

Comprehensive model fitting indicators, residual diagnostic test results, cross-validation performance, and 95% forecast uncertainty intervals for 2044 are summarized in Table 3 to fully demonstrate model robustness. Ljung-Box test results showed no significant residual autocorrelation for both prediction models, and ten-fold cross-validation yielded low MAPE values, indicating no overfitting and reliable long-term extrapolation performance.

**Table 3:** Model fitting, residual diagnostic and 2044 forecast indicators for OA burden time series.

Outcome indicator	Prediction model	R^2^	Ljung-box P-value	10-Fold cross-validation MAPE (%)	2044 predicted value (per 100,000)	95% prediction interval (per 100,000)
Age-standardized DALY rate	ARIMA (0,2,1)	0.999	0.723	1.21	292.02	(278.15, 306.79)
Age-standardized incidence rate	Brown exponential smoothing	0.997	0.681	1.56	589.00	(572.36, 608.42)

Note: MAPE, mean absolute percentage error; DALY, disability-adjusted life years. Ljung-Box *P* > 0.05 means residuals conform to white noise assumption without autocorrelation. Ten-fold cross-validation was applied to evaluate out-of-sample predictive stability and control overfitting. Wide 95% prediction intervals reflect inherent statistical uncertainty of 20-year long-term extrapolation.

**Fig. 5:**
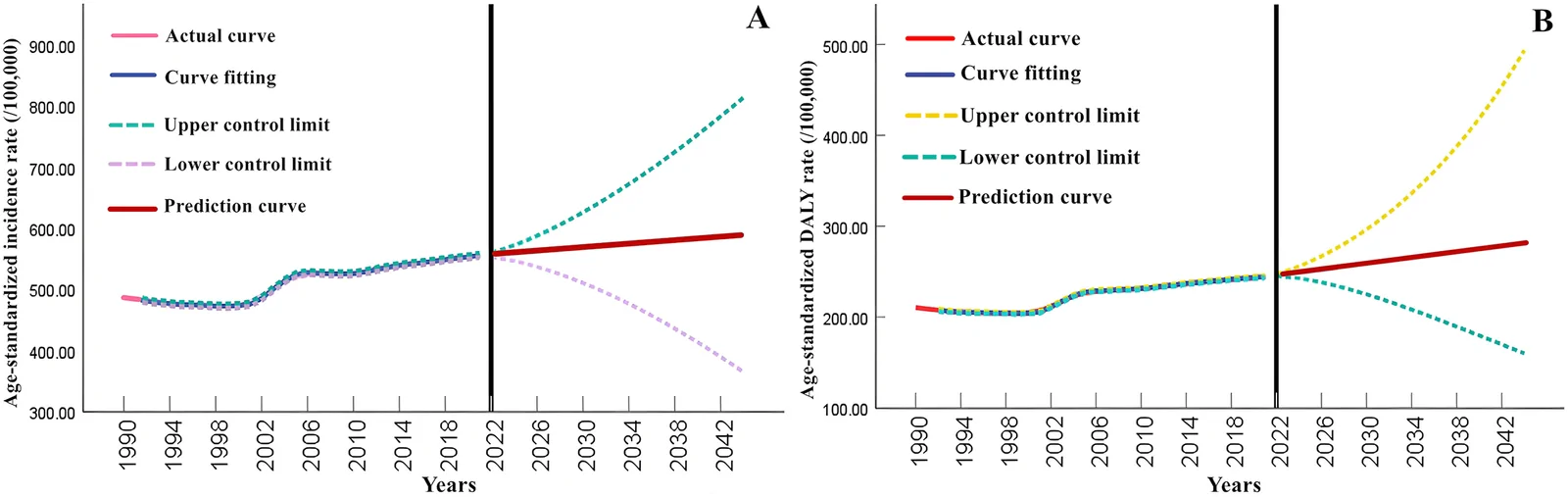
Projection of Osteoarthritis Disease Burden in China, 2025–2044. (A) The model of age-standardized incidence rate (/100,000). (B) The model of age-standardized DALY rate (/100,000).

Using the age-standardized incidence rate and DALY rate of OA from 1990 to 2021 as raw data, the optimal model for the standardized DALY rate was determined to be ARIMA (0, 2, 1) with an R^2^ of 0.999. The model projection showed that the standardized DALY rate of OA in China would continue to rise from 246.27 per 100,000 in 2022 to 292.02 per 100,000 in 2044. For the standardized incidence rate, the Brown model was selected with an R^2^ of 0.997, indicating an excellent fit to the historical trend. The projection indicated a steady upward trend, increasing from 556.11 per 100,000 in 2022 to 589.00 per 100,000 in 2044 (Fig. 5).

Uncertainty Consideration: Table 3 lists the 95% prediction intervals of all 2044 forecast indicators, with relatively wide interval ranges reflecting prominent statistical uncertainty for 20-year long-term extrapolation. While this study provides point estimates for future trends and the high R^2^ values (0.999 and 0.997) indicate good fitting for historical patterns, our univariate forecasting framework only relies on past OA burden data and cannot integrate dynamic future changes in population obesity levels, residents’ physical activity status and national healthcare policies. These key modifiable drivers may substantially alter actual OA trends and generate deviations between real-world burden and our predicted values, so all projection results must be interpreted prudently.

## 4 Discussion

In 2019, the global number of OA cases was approximately 527.8 million, and the number of OA patients in China reached about 100 million. Moreover, the disease burden of OA in China is still on the rise. Based on the GBD 2021 database, this study systematically analyzed the OA disease burden in China from 1990 to 2021 and projected its trend over the next 20 years. The results showed that the standardized DALY rate of OA in China was lower than the global level from 1990 to 2021; the incidence rate was lower than the global level from 1990 to 2003 but higher than the global level from 2003 to 2021. This upward shift after 2003 can be partially explained by population aging, rising national obesity prevalence and improved clinical diagnostic capacity; meanwhile, gradual healthcare system reform and enhanced outpatient medical access in China facilitated more OA case identification. Additionally, the OA disease burden in China showed an overall upward trend from 1990 to 2021, with the fastest growth in the first decade of the 21st century. The disease burden was significantly higher in females than in males, and the main affected joints were the knees and hands. Furthermore, projections indicate that the standardized incidence rate and DALY rate of OA in China will continue to rise steadily over the next 20 years, suggesting that OA control and management in China will face significant challenges in the coming decades.

The incidence rate and DALY rate of OA in China are significantly higher in females than in males, primarily due to physiological differences between genders. For example, estrogen may benefit the maintenance and health of cartilage and skeletal muscle through multiple pathways such as transforming growth factor β and insulin-like growth factor. After menopause, women's estrogen levels gradually decrease, affecting calcium absorption and utilization, thereby accelerating the degradation and wear of joint cartilage and increasing the risk of OA. In addition, low estrogen levels can induce inflammatory stressors, creating an inflammatory environment in the joints that leads to cartilage degradation and osteocyte apoptosis. On the other hand, weight gain during menarche, menopause, and pregnancy may contribute to a low-grade systemic inflammatory environment, which is another important risk factor for OA. The incidence rate of OA increases with age, and women generally have a longer life expectancy than men, resulting in a higher proportion of women in the elderly population—this may also explain the higher disease burden of OA in females. Apart from estrogen fluctuation and longer female life expectancy, divergent daily labor roles, varied occupational exposure and disparities in medical seeking behavior also contribute to gender-based OA disparity. However, the increase in OA disease burden is more pronounced in males than in females. This may be because Chinese males, as the main labor force of families and society, face higher joint loads in certain occupations such as heavy physical labor. Regardless of gender, the OA disease burden in China is still rising, and the situation is severe. Therefore, it is necessary to strengthen the popularization of OA-related knowledge, raise public awareness and attention to OA, advocate a healthy lifestyle (e.g., moderate exercise, avoiding prolonged poor posture), and encourage postmenopausal women to supplement calcium and vitamin D to maintain bone health.

OA incidence peaks at 55–59 years while DALY concentrates in people aged ≥85 years, with population aging as a core contributor [16]. In 2017, over 40% of China's population exceeded age 50 [17]; slow demographic growth under updated fertility policies [18] plus extended elderly life expectancy [19] will further expand the high-risk aging population. Shortage of specialized OA practitioners restricts standardized patient management [20], highlighting demands for policy optimization, routine elderly physical examinations and targeted health promotion.

Knee OA dominates disease burden due to lifelong high mechanical load and degenerative wear, followed by hand OA stemming from frequent daily joint use [21,22]. Contrary to global epidemiology, the strikingly low documented hip OA burden in China may be associated with insufficient screening and underdiagnosis, but this is merely an unvalidated hypothesis lacking clinical registry and imaging prevalence evidence rather than a definitive inference of true prevalence. In addition, inherent restrictions of the GBD database also restrict further refined stratified analysis for individual OA anatomical subtypes including knee OA. With no curative therapy available for OA, prevention should target verified high-risk cohorts: middle-aged women and knee OA susceptible populations, prioritizing modification of modifiable risks including obesity and acute joint injury with corresponding policy support.

As the GBD 2023 database has been newly updated, published global aggregate OA figures from GBD2023 show consistent upward secular trend of global OA burden compared with GBD2021, which indirectly supports our rising trend conclusion of China's OA burden based on GBD2021; detailed China-specific stratified 2023 OA data cannot be obtained at present, and incorporation of full GBD2023 Chinese sub-data will be prioritized in our follow-up continuous research.

## 5 Limitations

(i) No provincial-level stratified analysis was conducted in this study. Significant urban-rural disparities in medical resources and OA screening/diagnostic capacity exist nationwide. Rural areas lack sufficient imaging and primary care screening services, leading to underreported OA cases. As a result, the national aggregated burden trend calculated in this paper may underestimate the real OA prevalence and disability burden in remote rural populations. Subnational stratified burden evaluation will be included in our follow-up research.
(ii) OA data were extracted from the GBD 2021 database, which includes various data sources such as surveillance system data and individual-level survey data, meaning selection bias may affect the accuracy of OA disease burden estimates.
(iii) There are many factors affecting OA, which were not analyzed in detail in this study.
(IV) Our univariate ARIMA and Brown prediction models only extrapolate historical OA burden time series and cannot incorporate dynamic future variations in population obesity prevalence, physical activity levels and healthcare policy adjustments. These critical socioeconomic and behavioral determinants will continuously reshape the epidemiological trajectory of OA over the next two decades and may lead to notable forecasting bias. Meanwhile, the wide 95% prediction intervals of 2044 outcomes shown in Table 3 further demonstrate the high inherent uncertainty of 20-year extrapolation; all predicted values should not be regarded as definitive population estimates.
(V) This study cannot adopt GBD2023 China-specific detailed data due to restricted open access of country-level stratified indicators.

In conclusion, from 1990 to 2021, the incidence rate and DALY rate of OA in China showed an overall upward trend. The incidence rate and DALY rate were significantly higher in females than in males; the highest incidence rate was in the middle-aged population (55–59 years old), and the DALY rate was mainly concentrated in the elderly population. The huge burden of OA in China and its potential future growth indicate the need to develop a comprehensive OA management strategy. Currently, there are few safe and effective treatments for OA, and the main clinical management goals are to relieve pain and improve function. Therefore, primary prevention of OA may be more effective in reducing the disease burden [23,24]. For example, implementing public health education programs to emphasize the importance of maintaining a healthy lifestyle to reduce obesity risk, prevent knee joint injuries, and avoid strenuous joint-loading activities. Consistent with previous GBD-based burden research focusing on middle-aged and elderly Chinese populations [25], targeted population screening is essential to mitigate rising OA disability burden, especially regular knee OA screening for women aged 55–59 years. At the secondary prevention level, publicity should be strengthened to improve public awareness of OA. In addition, doctors should receive adequate training on OA treatment guidelines, such as exercise therapy to delay functional loss and appropriate use of analgesics to minimize side effects.

## Data availability statement

The raw data supporting the conclusions of this article will be made available by the authors, without undue reservation.

## Ethics statement

The studies involving humans were approved by the Ethics Committee of the 940th Hospital of Joint Logistics Support Force of Chinese People's Liberation Army. The studies were conducted in accordance with the local legislation and institutional requirements. Written informed consent for participation was not required from the participants or the participants' legal guardians/next of kin because this study solely extracted population public health-related data from open databases for statistical analysis and trend projection, no personally identifiable images or data are included in this study. Since the entire research process does not involve any intervention measures targeting human subjects, informed consent is not required.

## Author's contributions

Formal analysis: Zhe Wang.

Project administration: Chen Gao.

Supervision: Wantian Feng.

Writing – original draft: Binni Yang and Wantian Feng.

Writing – review & editing: Chen Gao and Zhe Wang.

All authors have read and agreed to the published version of the manuscript.

## Generative AI statement

The author(s) declare that no Generative AI was used in the creation of this manuscript.

## Funding

Not applicable.

## Conflict of interest

The authors declare no conflict of interest.
